# Cortical Gray Matter Injury in Encephalopathy of Prematurity: Link to Neurodevelopmental Disorders

**DOI:** 10.3389/fneur.2020.00575

**Published:** 2020-07-14

**Authors:** Bobbi Fleiss, Pierre Gressens, Helen B. Stolp

**Affiliations:** ^1^School of Health and Biomedical Sciences, RMIT University, Bundoora, VIC, Australia; ^2^Université de Paris, NeuroDiderot, Inserm, Paris, France; ^3^PremUP, Paris, France; ^4^Centre for the Developing Brain, School of Biomedical Engineering and Imaging Sciences, King's College London, London, United Kingdom; ^5^Comparative Biomedical Sciences, Royal Veterinary College, London, United Kingdom

**Keywords:** preterm, brain injury, development, inflammation, synaptopathy, MRI, functional activity, neuropathology

## Abstract

Preterm-born infants frequently suffer from an array of neurological damage, collectively termed encephalopathy of prematurity (EoP). They also have an increased risk of presenting with a neurodevelopmental disorder (e.g., autism spectrum disorder; attention deficit hyperactivity disorder) later in life. It is hypothesized that it is the gray matter injury to the cortex, in addition to white matter injury, in EoP that is responsible for the altered behavior and cognition in these individuals. However, although it is established that gray matter injury occurs in infants following preterm birth, the exact nature of these changes is not fully elucidated. Here we will review the current state of knowledge in this field, amalgamating data from both clinical and preclinical studies. This will be placed in the context of normal processes of developmental biology and the known pathophysiology of neurodevelopmental disorders. Novel diagnostic and therapeutic tactics required integration of this information so that in the future we can combine mechanism-based approaches with patient stratification to ensure the most efficacious and cost-effective clinical practice.

## Introduction

Preterm birth is defined as delivery before 37 completed weeks of gestation, and although the shorter the gestation, the higher risk of mortality and morbidity, even the late preterm-born infants are vulnerable to injury, including to the brain. The hallmarks of brain injury to the preterm born infant are: neuroinflammation, oligodendrocyte maturation arrest and hypomyelination, axonopathy, reduced fractional anisotropy and cortical volume determined by magnetic resonance imaging (MRI), and eventually, significant cognitive deficits (1). Collectively the brain damage associated with preterm birth is called encephalopathy of prematurity (EoP).

As long-term cognitive and behavioral consequences of preterm birth are increasingly recognized, neuropathological studies have focused on gray matter (GM), in addition to white matter (WM). It was initially thought that cortical GM injury only occurred in preterm infants in cases of very severe injury. Increased understanding of cortical development and more detailed post-mortem studies revealed that this not the case (2, 3). Over the past few years, work has increasingly indicated a widespread subtle neuronal injury in infants born preterm, that in some cases, such as interneuron deficits, may be independent of WM injury (4). Further than this, swathes of clinical and preclinical studies indicate that the cortical GM injury found in preterm infants significantly contributes to their increased risk of neurodevelopmental disorders (NNDs), such as autism spectrum disorder (ASD), attention deficit hyperactivity disorder (ADHD), and other learning and behavioral disorders.

The large and long running EPICURE (UK), EPIPAGE (France), and ELGAN (U.S.A.) studies have provided invaluable data on the incidence of neurological injury following very premature birth [e.g., (5–7)]. Together, these epidemiological studies confirm that preterm infants have a 25–30% incidence of neurological disorder, with as many as 40% of affected individuals having more than one diagnosable disorder (5–7). In all studies, incidence of cerebral palsy was 5–8% of preterm children, consistent between 2, 6, and 10 years of age (5, 6, 8). In addition, more than 40% of children at 2 years were below threshold for communication, motor, problem solving, and social skills (7), and 30% of children were diagnosed with cognitive impairment at 6 years of age (5), while at school age (10–11 years), 7–8% of preterm born children were diagnosed with ASD, 11% with ADHD, and 10% with emotional disorders, such as anxiety (8, 9). Using latent profile analysis in school age preterm born children (10 years of age), 25% of children were shown to have impaired executive functioning across a range of cognitive domains, while 41% of children fell into a “low-normal” category, where impairment was related to reasoning and working memory (10).

## Environmental Contributors to EoP and Mechanisms of Injury

The maternal fetal membranes surrounding the amniotic cavity represent the boundaries of a sort of “black box,” inside which we struggle to know and understand the processes preceding preterm birth. This is due to technical difficulties in safely monitoring the biochemical processes ongoing in the uterine space. However, processes causing brain injury in the preterm born infants certainly begin before delivery, as indicated by a small study of brain functional connectivity in fetuses who went on to be born preterm (11) and an increasingly number of studies showing predictive biomarkers in maternal blood months before preterm birth (12–14). Numerous events and antenatal exposures have also been associated with preterm birth and EoP via epidemiological study and verified with preclinical studies. These include placental abruption or twin–twin transfusion, preeclampsia, or placental insufficiency (potentially contributing to a hypoxic-ischemic-like insult and/or intrauterine growth retardation) and, less commonly, complications linked to oligohydramnios and maternal substance abuse (15). A predominant role of hypoxia in EoP with no other complications (such as those described above) is not supported by clinical data (16). Chorioamnionitis, leading to a maternal–fetal inflammatory response, is a chief driver of the process of early parturition leading to preterm birth, demonstrated across clinical and preclinical studies (17–19). Maternal–fetal inflammatory response not only precipitates preterm birth, but a wealth of epidemiological and clinical studies have shown that, although it is often clinically silent, it is a major driver of EoP (20, 21) and its associated long-term neurological and behavioral/psychiatric deficits [see reviews (22, 23)]. While EoP can be initiated prenatally, there is evidence of continued disruption of the brain post-natally, which could be driven by a mixture of pre- and post-natal factors. For instance, Bouyssi-Kobar et al. (24) show reduced brain growth trajectories in preterm born infants compared to age-matched *in utero* controls that were associated with (antenatal) chorioamnionitis, as well as post-natal sepsis. Inflammatory drivers include pre- and post-natal events and conditions: chorioamnionitis, funisitis, early and late onset sepsis, and necrotizing enterocolitis. Other, non-inflammatory, post-natal contributors to EoP may include hyperoxia (25), as the *ex utero* environment is relatively high in oxygen compared to the *in utero* environment (26), and reduced exposure to maternal hormones, such as estrogen and other neuroactive precursors (27). While there has been little follow up on the estrogen hypothesis clinically (28), recent animal models have suggested a potential protective effect (4, 29, 30). That hyperoxia plays a role in EoP is also supported by animal studies (31–33).

## Cellular Mediators of Brain Injury

How, specifically, do these perinatal events lead to EoP? In the case of maternal–fetal inflammation, systemic inflammation drives changes in the brain after either crossing directly through the endothelial cells making up the blood–brain barrier (BBB) or by stimulating, via receptors for cytokines, such as interleukin-1 (IL-1), production of pro-inflammatory molecules by the endothelial cells that are secreted into the brain parenchyma (34, 35). It is currently unclear whether these immune mediators act directly on neurons or have their actions performed via stimulated glial cells, such as microglia and astrocytes, though it is likely that both processes occur. Inflammation has been shown in the fetal brain to reduce neurogenesis in embryonic proliferative zones (36) or to increase neurogenesis in the SVZ and dentate gyrus (37), which has been linked to microglia activity in some cases [reviewed in (38–40)]. The timing of the exposure of neurons to inflammatory stimuli is undoubtedly critical. The exact timing of events impacting EoP is not clear, though it is likely that events occurring in the third trimester are most influential. Although the vast amount of proliferation is complete in the third trimester, there are specific cell types, interneurons of note, that are still being born and migrating in this period and that are increasingly demonstrated to be vulnerable in preterm born infants and animal models (41, 42). Susceptibility of processes, such as neuronal arborization and synapse formation may be even more important, given the intersect between injury events sensitizing to EoP and the developmental timetable of the brain (discussed in more detail below).

Over the past decade, the importance of microglial activation has been exhaustively demonstrated in human preterm-born infant post-mortem brain samples and in models of perinatal brain injury [reviewed in (43, 44)]. These studies have included experimental evidence that microglia are necessary for the evolution of injury in the developing brain (45, 46), but conversely, that microglia also play protective roles in perinatal brain injury (47, 48). Microglia are also activated by other modulators of brain injury, such as hypoxia or hypoxia-ischemia, as these events lead to cell injury and the release of damage-associated messenger proteins (DAMPS) and/or the production of toxic metabolites that also activate microglia directly (49, 50). Microglia establish territories in the developing brain from early in embryonic life and are intimately involved in the processes of brain building. Thus, microglia activation to an immune responsive state leads to EoP via a double hit, whereby there is production of toxic factors for neighboring neural cells and loss of normal microglial functions to shape axonal connectivity and synaptic elimination/function. This has been well-described in the WM (51–54). There is substantially less information on the specific effects of activated microglia on the GM, including synapses and interneurons, which are relevant to EoP. However, as increasing evidence shows that microglial dysfunction persists for weeks to years after insult (55–58), the importance of this phenomena may become more apparent with further study. It is also plausible that the GM and WM are differentially vulnerable, as microglia in these tissues have differing gene expression patterns in the basal state and after injury, based on studies in adults (59, 60). However, nothing is known of this difference in a model relevant to EoP. As such, although we can speculate that there may be specific soluble factors or regulators (micoRNAs, cytokines, etc.) released (possible via vesicles) from GM microglia that influence GM development in ways that would offer therapeutic avenues (61, 62), evidence is still required.

Reactive astrogliosis is also observed in some forms of human perinatal WM injury (63, 64) and is associated with deleterious effects mediated by agents, including hyaluronic acid (65), bone morphogenic protein (66), cycloxygenase-2 induction and associated prostaglandin E2 production (64), and endothelin-1 (67), which can impair oligodendrocyte precursor cell maturation. Clinical and experimental studies have shown a role for GFAP-positive astrocytes in WM injury in preterm born infants, but specifically in older preterm born infants [>32 weeks; (68, 69)] and during equivalent stages of rodent development [5 post-natal days plus; (70)]. In the GM, astrocytes increase in number with gestational age. Compared to the WM, GFAP positive cells represent a far smaller proportion of cells (<1%) (71) and the response of the populations a whole to injury is under-studied. In preterm-born infants, a small increase in the number of GM astrocytes was reported in a study of infants with cystic periventricular leukomalacia (cPVL; severe injury), but as the control group had a significantly lower gestational age, this effect did not survive correcting for development (72). However, studies in animals support the hypothesis that astrocytic dysfunction proceeds neuronal damage in at least some injury paradigms (73).

## Developmental Events Susceptible to Injury in the Preterm Brain

When assessing how pre- and post-natal factors contribute to EoP, it is necessary to consider what developmental events happen during the preterm period that may be affected by preterm birth. The prenatal period most associated with EoP (from 23 to 32 weeks) is characterized by the final stages of neurogenesis in the human telencephalon, neuronal migration, differentiation and maturation, and the very early stages of cortical myelination. Neurogenesis peaks very early in gestation (8–12 weeks), but continues both in the ventricular zone of the dorsal cortex and within the ganglionic eminences for up to 29 weeks (41). The cortical plate forms around 11 weeks into gestation [reviewed in (74)] until shortly after the end of neurogenesis. Excitatory neurons primarily come from the progenitors in the ventricular zone of the dorsal cortex and migrate radially to the cortical plate [reviewed in (74)], while inhibitory cortical neurons derive from the ganglionic eminences and migrate tangentially to the cortical plate [reviewed in (75)]. Once neurons reach their final positions in the cortical plate, they start to mature—a process which includes extending dendritic arbors and forming synapses, detectable form 19–23 weeks gestation (76). At the same time, radial projections of the neural progenitors are lost, and tangential extension of subcortical and cortico-cortical projections continues. These processes continue through the prenatal and post-natal period of brain development, with an extensive period of synaptic modulation and pruning throughout the first year of life. Local electrical activity and connectivity between neurons can be detected early, undergoing numerous changes over development, and don't appear to find a mature state until early adolescence [reviewed by (77)]. Details of these events and many of the mechanisms underlying them are reviewed extensively in Molnar et al. (78) and Volpe (79). On top of these microstructural changes is a general increase in cortical thickness and a semi-stereotypical pattern of cortical folding, with primary sulci forming from 16 to 19 weeks gestation, and secondary and tertiary sulci formation starting from 32 to 36 weeks, respectively (80).

The real-time development of the brain, including the increasing complexity in the cortical structure, can be detected with non-invasive imaging methods, such as T1/T2 or diffusion-weighted MRI [(81–83); reviewed by (84)] and these techniques have allowed the detection of delayed or impaired cortical development in preterm born infants. However, given the relatively low resolution and integrated nature of diffusion MRI signal, interpreting the specific structural changes in relation to neurodevelopmental processes is difficult. Techniques are in development to scale match MRI and histological data [e.g., (85)], though changes in diffusion MRI are currently interpreted through comparison with standard histological measures. There is evidence that preterm birth can result in changes to all the processes of cortical development described above, including reduced progenitor proliferation, arborization, and myelination, as well as direct injury outcomes, such as cell death. The rest of this review will discuss a number of these in detail, including potential mechanisms of injury, overlap with neurodevelopmental disorders (NDDs), and potential therapeutics.

## Macroscale Alterations in Cortical GM

Many elegant neuroimaging studies have begun to correlate both typical and pathological behavior with specific brain areas or functional readouts. Based on these, we know that many brain regions contribute to the diverse array of neurological disorders presented by preterm-born children. In particular, the important role of cortical dysfunction underlying these cognitive disorders (but not so prominently in motor disorders) is increasingly clear. Rathbone et al. (86), in their study of cortical growth (cerebral volume and cortical surface area) in the 20 weeks between birth and term-equivalent age in preterm infants, showed that slow rates of cortical growth correlated directly with neurocognitive ability at 2 and 6 years of age. In particular, impaired cortical surface area growth correlated with poorer scores in numerous features of executive function, learning, memory, and attention, as well as social ability. However, there was a clear lack of correlation between cortical growth and motor abilities (86). Numerous neuroimaging studies using different post-imaging assessment methods have shown reductions in cortical GM volumes in preterm infants, both in the preterm population as a whole (87–89) or specifically those with periventricular leukomalacia [PVL; (90, 91)] and in very preterm born children [assessed at 7 years; (92)] and adolescence [assessed at 15 years; (93, 94)]. Reductions in volume of the deep GM have also been reported (95, 96); changes in thalamic volume were found to be a predictor of reduced cortical GM volume and alterations in diffusivity within the thalamocortical networks (95) were found to correlate with cognitive performance at 2 years of age, though they only accounted for 11% of the variance (97).

Importantly, Bora et al. (98) showed that very preterm infants had a 13%-increased risk of inattention and hyperactivity behavior at school age (4, 6, and 9 years), which correlated with decreased GM volumes, particularly within the prefrontal region. Increased anxiety-like behavior has also been associated with preterm born infants with reduced GM volume (99). In a small study group, very preterm infants that went on to have a diagnosis of ASD were found to have increased incidence of cystic WM lesions, and reduced cerebellar volume, but no changes in GM volume (100). However, only eight children in the cohort (4.7%) were diagnosed with ASD by the age of 7 years, so the study may have been underpowered for detecting more subtle changes in cortical GM.

In animal models, mimicking changes in these cortical volume parameters is difficult, due to differences in the relative GM and WM volumes in experimental species and the differences in the size/complexity of the individual regions relative to one another. Sheep are used in studies of perinatal brain injury, with advantages regarding physiological and neurological similarities to preterm humans, including gray–white ratios [discussed in (101)]. Dean et al. (102) have studied intra-amniotic LPS in sheep, a paradigm able to cause cystic WM injury. This inflammatory exposure caused no obvious cell loss in the GM, but reduced cortical volume by ~18%. In further work in sheep models, Dean et al. (103) also showed a reduction in cortical GM volume after *in utero* hypoxia-ischemia, in which there was again no cortical cell loss. Interestingly, there was no early reduction in cortical volume (+7 days) but these became increasingly apparent with time after injury [starting at 2 weeks and at least up to 4 weeks; (103)]. In a mouse model of maternal immune activation using poly I:C, subtle decreases in GM volumes were observed throughout development (104), though changes in cortical volumes were not specifically reported.

In addition to alterations in GM volume, complex changes in cortical architecture have also been identified. For instance, Zhang et al. (92) determined that there was a decrease in cortical surface area and the gyrification index of 7-years-old following very preterm birth compared to term-born controls. Maps of cortical folding patterns in neonates suggest that preterm infants have fewer and shallower sulci than term equivalent controls (105). Data suggested that the lower gyrification index and cortical surface area in preterm-born neonates was likely to be due to a combination of altered *in utero* and post-natal growth, and it was a finding independent of reduced total brain volume (105, 106). Reduced cortical folding has also been associated with increased incidence of NDDs later in life (106), matching, at least partly with data from specific disorder cohorts (107–110), though the data are not substantial here, as existing studies are small.

Collectively, these data point to the possibility that alterations in cortical folding are driven by numerous age-specific microstructural changes. The theories behind cortical folding are many, and include processes such as the rate of neurogenesis, of tangential migration and neuronal arborization [reviewed by (111–113)]; currently no single one is sufficient to explain the biological underpinning to normal or abnormal cortical folding. Numerous aspects of the *in utero* environment and preterm injury have been associated with changes in cortical folding [reviewed by (114)], though the mechanism by which this injury is produced is still unclear. Recent work by Garcia et al. (115) has shown regional differences in cortical growth rate between post-menstrual age 30 and term equivalent age (based on 2–4 MRI scans over this period in preterm-born infants), which are disrupted in preterm-born infants with gross injury, such as intraventricular hemorrhage (IVH). Their work suggests that severe injury in preterm born infants may alter local cortical growth and subsequently cortical folding (115), supporting the hypothesis that cortical folding may result from mechanical instability as the GM grows faster than underlying WM (116). Alternatively, recent compelling evidence also shows that the extracellular matrix is essential in normal cortical folding (117), likely contributing to the mechanical tension within the brain. The link between these highly reductionist *ex vivo* studies and EoP is currently unclear, though they have suggested that hyaluronic acid can inhibit cortical folding (117), and increases in hyaluronic acid have been found within the preterm brain (118).

## Gray Matter Neuropathology Associated With EoP

There are few neuropathological studies of GM injury in preterm-born infants, compared to the number of studies of WM injury. Complicating matters, due to the difficulties of defining appropriate controls, GM studies typically use evidence of WM injury as a starting point in the assessment of the GM, rather than searching for independent patterns of injury. We have created Table 1, which summarizes studies performed on human preterm-born, post-mortem tissue that have included GM analyses. From this, we can generalize that in studies of infants with severe and contemporaneously uncommon WM injury (cystic PVL), there are reductions in neuronal number and increased neuronal cell death (where assessed; 6/6 studies). However, in studies of infants predominantly with diffuse WM injury, global reductions in cell number are less frequently reported (1/5 studies), but interneurons seem to be a vulnerable subpopulation (present in 3/3 studies) and dysmaturation in cerebellar lamination are reported (1 study). Interestingly, there are complex subtle changes in interneurons in cases with non-cystic WM injury vs. controls (42) and when comparing very preterm infants to less preterm infants [irrespective of WM injury; (4)]. It is necessary to note that of the 12 studies identified, 6 of these were performed on archival tissue collected between 1993 and 2007 from the Department of Pathology at the Children's Hospital Boston. An additional observation study was not included in the table, as the data were expanded upon in a later study (127). It is not possible from the published details of the Boston group's work to determine whether cases in these studies have been reused. Thus, reports of cell death across regions in these studies may be interdependent, due to case severity in this center, and studies of other centers and in more contemporaneous cohorts are needed to determine the state of neuronal injury in preterm born infants more generally.

**Table 1:** Summary of post-mortem studies of preterm-born infants, including analysis of the GM, highlighting the case characteristics, regions of interest, and the GM and WM injuries described.

**Reference** / ***Study location*** / **Years of sample collection**	**Number of cases (n)** / ***Gestational age at birth*** / **Survival time**	**Pathologists description of injury** / ***Post-mortem delay***	**Nature of cases designated as controls**	**Regions of interest**	**Gray matter pathology (description of what was analyzed)**	**White matter pathology (description of what was analyzed)**
Andiman et al. (119) / *Dept. of Pathology, Children's Hospital Boston, MA, USA* / **1993–2007**	20 WMI, 15 controls / *WMI = 33.9 ± 4.3, Control = 33.1 ± 6.2* / **WMI = 5.9 ± 14.0 weeks, Controls = 13.2 ± 23.6 (NS diff)**	PVL or diffuse WMI as diagnosed by a histopathologist / *Post-mortem delay not reported*	Prematurity with respiratory distress syndrome, *n* = 4; congenital heart disease, *n* = 2; primary pulmonary hypertension, *n* = 1; hydrops fetalis due to placental chorioangiomas, *n* = 1; hydrops fetalis, *n* = 1; sacral teratoma, *n* = 1; cystic lymphatic malformation of the neck, *n* = 1; Werdnig–Hoffmann disease, *n* = 1; foreign body aspiration, *n* = 1; Blackfan–Diamond syndrome, *n* = 1 and bronchiolitis, *n* = 1 No difference in mean Apgar scores at 5 min (6.8 for both groups) or in other disorders / confounders	In the WMI cases, the cortex overlying WMI and compared it to similar cortical areas in control cases	No sig. difference in the presence of fractin-immunopositive neurons in any cortical layer No sig. difference in the incidence of the percent of MAP2-stained pyramidal cells in layer V or obvious cortical anomalies. Significant reduction (67%) in the density (MAP2) of layer V pyramidal neurons No sig. difference in the cortical or laminar thickness (MAP2, H&E)	Periventricular focal necrosis in the deep white matter with surrounding diffuse reactive gliosis and microglial activation (previous neuropathologic studies)
Haynes and van Leyen (120) 12 / 15-Lipoxygenase / *Dept. of Pathology, Children's Hospital Boston, MA, USA* / **Collection epoch not reported**	13 PVL, 17 controls / *PVL −29 to 43 PC weeks (median = 35.5) and 0–8 PN weeks (median = 1.5), Controls −20 to 43 PC weeks (median = 33.5) and 0–2.5 PN weeks (median = 1)* / **Survival time not reported**	PVL as diagnosed by a histopathologist / *PVL = 6–25 h, Control = 4–25 h*	Control cases did not have PVL or other significant brain pathology upon standard histologic examination. Autopsy reports were reviewed for major clinical findings, systemic autopsy diagnoses, and neuropathologic findings	Subcortical white matter and the cortex overlying WMI and compared it to similar cortical areas in control cases	No increase in 12/15-LOX expression in neurons of the cerebral cortex in PVL. Cell death or total cell number not assessed in the gray matter	PVL had “focal” necrotic component in the periventricular region, and “diffuse” component characterized by reactive gliosis and activated microglia in the surrounding white matter Increased 12/15-LOX expression in large round CD68^+^ cells, lectin double positive and O4 double positive cells and scattered TUNEL- positive cells
Haynes et al. (121) Diffuse axonal / *Dept. of Pathology, Children's Hospital Boston, MA* / **Collection epoch not reported**	13 PVL, 17 Control (spread across acute and later stages) / *Mean gestational age (wks) PVL =36 ± 3, Controls = 32 ± 7* / **Mean postnatal age (wks)—PVL = 7.5 ± 17, Control = 10.5 ± 27**	PVL as diagnosed by a histopathologist / *PVL = 6–44 h (median = 17), Controls = 1.5–132 h (median = 14)*	Control cases did not have PVL or other significant brain pathology upon standard histologic examination. Causes of death included Noonan's syndrome 1, Fetal hydrops 1, Neonatal hepatic disease 1, Immune thrombocytopenia 1, Possible mitochondrial disorder 1, Sudden unexplained death in childhood, 1, Trisomy 21 1, Unexplained stillbirth	The area of study for axonal damage in PVL was distant from the infarct, i.e., in a separate section with no overlying cortical damage	Approximately 1/3rd PVL cases had thalamic gliosis, neuronal loss, and / or microinfarcts as determined by conventional histopathologic examination. Visually appreciable neuronal loss was present in the overlying cerebral cortex in 15% of the PVL cases. None of the non-PVL, non-axonal controls examined showed evidence of thalamic and / or cerebral cortical damage	PVL based on histopathologic criteria—periventricular focal necrosis in association with diffuse reactive gliosis and microglial activation Diffuse axonal injury, as determined by the apoptotic marker fraction, in the gliotic (non-necrotic) cerebral white matter in the acute and organizing stages of focal PVL
Ligam et al. (122) / *Dept. of Pathology, Children's Hospital Boston, MA, USA* / **Collection epoch not reported**	22 PVL, 16 non-PVL / *Gestational age in PVL = 32.5 ± 4.8 gw, Controls = 36.7 ± 5.2 gw, Sig dif in gw*. / **PVL = ~4 weeks, Controls = ~20 weeks (*****P*** **= 0.07)**	PVL as diagnosed by a histopathologist / *Post-mortem delay not described*	Control cases did not demonstrate white matter features. Lower rates of NEC and sepsis in controls than in PVL	Thalamic sections were analyzed at one of the following levels: I (anterior), level of the mammillary bodies; II (mid), level of the red nucleus; and III (posterior), level of the lateral geniculate nucleus	Increased thalamic pathology via neuropathologist assessment (H&E) Trend to decreased neuronal density with H&E (*p* = 0.07)—criteria for neuronal discrimination not described Increased density of reactive astrocytes (GFAP) in the mediodorsal nucleus and the lateral posterior nucleus No significant increase in the density of CD68 + cells and numbers overall low No difference in the density of MDA-immunopositive neurons or percent of MDA-immunopositive neurons	Histopathology to confirm (or not) PVL, with “focal” necrotic component in the periventricular region, and “diffuse” component characterized by reactive gliosis and activated microglia in the surrounding white matter
Kinney et al. (123) / *Dept. of Pathology, Children's Hospital Boston, MA, USA* / **1998–2012**	15 PVL, 10 controls / *The mean gestational age PVL = 32.8 ± 4.1 weeks in the, Control = 30.1 ± 5.9 weeks* / **PVL = 34 ± 4.6 postconceptional weeks, Controls = 31.6 ± 6.6 postconceptional weeks**	PVL as diagnosed by a histopathologist / *Causes of death in PVL: respiratory distress syndrome (*n* = 7); congenital heart disease (*n* = 3); primary skeletal disorders (*n* = 2); congenital diaphragmatic hernia (*n* = 1); inborn error of metabolism (*n* = 1) and VOGM (*n* = 1)* / *PVL = median 14 h, Control = median 16.5 h*	Controls did not demonstrate white matter abnormalities Causes of death in controls respiratory distress syndrome (*n* = 5); congenital heart disease (*n* = 1); hydrops fetalis due to placental chorioangiomas (*n* = 1); hydrops fetalis due to parvovirus (*n* = 1); primary pulmonary hypertension (*n* = 1); and bronchiolitis (*n* = 1)	Neurons in the ventricular/ subventricular region, periventricular white matter, central white matter, and subplate region in PVL cases and controls—including five subtypes of subcortical neurons: granular, unipolar, bipolar, inverted pyramidal, and multipolar	The neuronal density of the granular neurons in each of the four regions was 54–80% lower (*p* ≤ 0.01) in the PVL cases compared to controls adjusted for age and post-mortem interval The overall densities of unipolar, bipolar, multipolar, and inverted pyramidal neurons did not differ significantly between the PVL cases and controls	Analysis grouped neurons in the subplate and white matter collectively PVL was characterized by necrotic foci in the periventricular and/ or central white matter; and diffuse astrogliosis and microglial activation in the surrounding white matter
Pierson et al. (72) / *Dept. of Pathology, Children's Hospital Boston, MA, USA* / **1997–1999**	17 PVL, 17 DWMI, 7 Negative (controls) / *PVL = 3.7 ± 4.1 (median = 2.3), DWMI = 3.4 ± 14.0 (median = 1.2), Negative = 0.8 ± 1.2 (*n* = number of samples)*	PVL or diffuse white matter gliosis (DWMG) without necrosis / *Post-mortem delay not described*	“Negative” white matter group with no diffuse gliosis or focal periventricular necrosis in the cerebral white matter	Seventeen gray matter regions, across the limbic system, cerebral cortex, deep gray nuclei, cerebellum and relay nuclei Seven white matter regions—frontal lobe, temporal lobe, parietal lobe, occipital lobe, corpus callosum, internal capsule and cerebellum	Sig increased neuronal injury in the cerebellar cortex and frontal cortex of PVL compared with DWMI or Negatives (H&E) No increase in astrogliosis (GFAP)	Focal periventricular necrosis; diffuse white matter gliosis
Haldipur et al. (124) / *National Brain Research Centre, Manesar, India* / **2007–2010**	40 cases / *Across the window of 28 weeks of gestation to 8 postnatal months* / **4 controls with 0 days survival and 32 cases of varying age at birth and survival**	All cases are those in which the autopsy indicated minimum or no damage to the brain and cerebellum in particular / *Delay = <24 h*	Still birth cases—with no obvious signs of injury as per cases with postnatal survival	Cerebellum	EGL cell density significantly increased by preterm birth EGL thickness reduced by preterm birth	None described
Marin-Padilla (3) / *Paediatric Autopsy Service, Dartmouth-Hitchcock Medical Center, Hannover, New Hampshire, USA (via ref 23)* / **Collection epoch not reported**	33 cases total / *5 cases born preterm who all had short survival time* / **3 months through to 5 years survival**	PVL as diagnosed by a histopathologist / *Post-mortem delay not described*	No controls—description of changes over time after WM injury only	Gray matter overlying frank WM injury	No changes visible in the acute cases—which were the pre-term born infants In the cases surviving longer—late term and term born infants, no change in the upper cortical layer vascular and cellular distribution and morphology (H&E, Golgi) Axomatoised pyramidal neurons change from being long projecting to being local-circuit (Golgi). These cells have increased circuitry and altered neuronal morphology—populations of larger and smaller cells with altered distributions (Golgi, H&E, GFAP)	Cystic white matter lesions
Stolp et al. (42) / *Perinatal Pathology Department, Imperial Health Care Trust, London*,	Non-WMI group, *n* = 7, WMI group, *n* = 6 / *Non-WMI group = 23 + 2 to 28 + 1 gw, WMI group 26 + 5 to 29 + 3*	Evidence of diffuse (non-cystic) white matter injury (WMI cases) including white matter gliosis and focal lesions / *1–3 days—bodies stored at 4°C*	Seven cases showed no significant brain pathology, non-neuropathologic controls (no WMI cases)	Interneurons of the frontal cortex and underlying white matter	No change in the total number of cortical neurons, identified by HuC/ HuD immunoreactivity, with 53,104 ± 11,009 immunopositive cells/ mm2 found in the control brains (*n* = 5)	No statistical differences in the number of SST or NPY subpopulations in the white matter between preterm infants with or without white matter injury. Significant decrease in the
*UK* / **Collection epoch not reported**	*gw* / **Non-WMI group = 5 min to 43 h. WMI group 1 min to 5 weeks (comparison** ***p*** **= 0.002)**				and 52,120 ± 6,327 cells/mm2 in the cortex of the white matter injury cases (*n* = 4) Significant decrease in the cortical calretinin+ cells Calbindin- and parvalbumin-positive cells were observed in low numbers in both cases, insufficient for determining statistically significant changes. Somatostatin and Neuropeptide Y only found in the white matter	arborization of Somatostatin and Neuropeptide Y interneurons in both of these interneuron classes As previously reported (125)
Panda et al. (4) / *New York Medical College and Albert Einstein College of Medicine, USA* / **2002–2016**	Fetuses: 20–22 gw, *n* = 5, Infants: 23–28 gw, *n* = 5, Inafnat: 29–34 gw, *n* = 5 / *20–40 gestational weeks (gw): 26–27 gw infants surviving for 4–6 weeks were compared with 32–33 gw infants who lived for ~3 days. Therefore, both had PMA33 gw at their death*	Excluded = moderate to severe intraventricular hemorrhage, major congenital anomalies, history of neurogenetic disorder, chromosomal defects, culture-proven sepsis, meningitis, or hypoxic–ischemic encephalopathy and infants receiving extracorporeal membrane oxygenation treatment / *post-mortem interval of ~18 h*	None. Comparisons of effects of varying degrees of prematurity	Cortex (cortical plate), white matter (embryonic intermediate layer), and ganglionic eminences, which were cut at the level of the head of caudate nucleus	More prematurely born infants have fewer GAD67+ neurons in upper and not lower cortical layers More prematurely born infants have fewer Parvalbumin+ neurons in upper and not lower cortical layers More prematurely born infants have greater numbers of Somatostatin+ neurons in upper and not lower cortical layers Calretinin+ and neuropeptide Y+ interneurons not effected by preterm birth	No analysis undertaken.
Vontell et al. (125) / *Perinatal Pathology Department, Imperial Health Care Trust, London, UK* / **Collection epoch not reported**	7 WMI and 7 controls / *All <32 weeks' gestational age, vaginally delivered* / **Survival time not reported**	Cerebral white matter gliosis, lipid-laden macrophages, and focal lesions with evidence of WMI on pathologic examination (WMI cases) / *1–3 days—bodies stored at 4°C*	Also, extremely preterm, but with no significant brain pathology on gross and microscopic examination from post-mortem examination and had no visible brain abnormalities on post-mortem magnetic resonance imaging	Thalamus (medial dorsal (MD) nucleus, ventral lateral posterior (VLp) nucleus, ventral posterior lateral (VPL) nucleus) White matter [posterior limb of the internal capsule (PLIC) adjacent to the VLp (PLIC-VLp) and PLIC adjacent to the VPL (PLIC-VPL)]	No difference in the total average cell density in thalamic regions (H&E) Significant decrease in neurons in WMI cases in the MD, VLp, and VPL (HuC/ HuD) Significant increase in the ratio of astrocytes (GFAP+) to total cell count in thalamic regions in WMI cases, compared with MD (*p* < 0.01) and VLp (*p* < 0.01)—but not VPL Significant increase in IBA1+ cells in WMI cases in the MD, VLp, and VPL.	No difference in the average total cell density in white matter regions Significant increases in neurons in PLIC-VPL but not in PLIC-VLp Significant increase in IBA1+ cells in the PLIC-VPL (*p* < 0.05), but not in PLIC-VLp
Pogledic et al. (126) / *Hôpital Robert Debré, Paris, France* / **Collection epoch not reported**	Cystic (c)-WMI, *n* = 7, Controls, *n* = 18 / *c-WMI = 24 + 4 to 27 gw, controls = 24 + 2 to 34 gw* / **c-WMI = 0–4 weeks and 1 day, Control = 0–11 days**	Cystic cases including focal lesions with macroscopic cysts associated or not with necrosis and / or calcifications surrounded by diffuse pallor / *post-mortem interval <48 h*	Non-cystic cases without tissue loss displayed pallor and gliosis (18/18 cases) associated with microscopic necrotic foci in a few cases (4/18 cases) and were considered to consist of diffuse lesions	Cortical regions located in the posterior part of the superior, middle and inferior frontal gyri and sulci, and the precentral gyrus and central sulcus corresponding to corresponding to the presumptive premotor and motor areas (areas 8-6-4) and contiguous prefrontal areas	Significantly increased cortical plate and subplate astrogliosis (GFAP) in c-WMI vs. control preterm WMI (no change in very preterm cystic and control cases) No increase in cortical plate and subplate microgliosis (IBA1) in c-WMI vs. control preterm WMI (for very preterm or just preterm cases)	White matter cysts were confined to the white matter without extending into superficial layers of the cerebral wall, such as the subplate and cortical plate

*WM injury provided as context for overall injury severity*.

*Of the 12 studies identified, those highlighted in yellow (n = 6) report studies performed on tissues drawn from the same pool of post-mortem samples between the years of 1993–2007. It is unclear, and undeterminable from the case reports, how many times a single case appears across the six studies, and as such, how co-dependent the findings are. 12/15-LOX, 12/15-lipoxygenase; DWMI, diffuse white matter injury; EGL, external granule cell layer; h, hours; GAD67, glutamate decarboxylase 67; GFAP, glial fibrillary protein; gw, gestational weeks; H&E, hematoxylin and eosin; MAP2, microtubule associated protein 2; MD, Medial dorsal nucleus (thalamus); MDA, malondialdehyde; NEC, necrotising enterocolitis; PC, post-conceptional; PLIC, posterior limb of the internal capsule; PVL, periventricular leukomalacia; TUNEL, terminal deoxynucleotidyl transferase dUTP nick end labeling; WMI, white matter injury; VLp, ventral lateral, posterior (thalamus); vPL, Ventral posterolateral nucleus (thalamus)*.

## Animal Models of GM Pathology

### Severe Injury

Severe brain injury, including cystic lesions and severe IVH, occurs in very few preterm-born infants [<5% cystic lesions, <5% IVH; (128)]. Historically, the proportion of infants with these forms of injury was much higher. It was also once considered that hypoxia-ischemia was the leading (possibly the predominant) cause of perinatal injury, including in preterm-born infants. Because of these historical trends and (now updated) ideas, much of the data that we have on GM injury in EoP is from animal models of gross clastic lesion (30–80% hemispheric ablation). This initial wave of data suggested that of the cortical layers, the subplate was most susceptible to hypoxic-ischemic injury at preterm equivalent ages (129), possibly due to its relatively early birth and maturation. However, subsequent studies agree that the extent of cortical injury is dependent on the severity of the insult, and all lower cortical layers have the capacity for cell loss after severe hypoxia-ischemia (130). In a study of partial uterine artery occlusion, modeling hypoxia-ischemia in the fetal sheep, immediate low level necrotic cell death was found throughout the deep and cortical GM, followed by extensive apoptosis in both the GM and WM at 3 h post-injury (131). Other studies of *in utero* hypoxia-ischemia in sheep have shown some increase in pyknotic cells and activated caspase-3 staining from 24 h up to 4 weeks in the caudate nucleus and subplate (132, 133), reduced NeuN and somatostatin positive neurons in the caudate and putamen (134), specific loss of glutamate decarboxylase interneurons (a marker of GABAergic neurons) and their perineuronal nets in the cortex (135), along with reduced arborization complexity and spine density in both the caudate, hippocampus, and cortex (103, 132–134, 136).

### Moderate/Mild Injury

Improvements in antenatal and post-natal care, including the use of prenatal steroids and post-natal surfactants and improved respiratory support, have collectively led to the decrease in severe brain injury, so that now the vast majority of infants suffer from diffuse WM injury (118). This has inspired the revision/creation of animal models focused on modeling white matter dysmaturation. A number of these new(er) models providing insights into the role of contemporaneously relevant insults to GM injury are described. A landmark study in our understanding of the GM injury induced by preterm birth came from the team led by Sandra Rees (137), wherein they delivered baboons preterm and held them in a NICU environment for 2 weeks. This important study isolated the roles of prematurity itself from exogenous/precipitating factors (such as chorioamnionitis and sepsis). WM injury and hemorrhage were most common in preterm baboons, but there were significant pockets of necrosis in layer IV/V cortical neurons (4/16 cases) and in the head of the caudate (1/16 cases). One of the first attempts to nail down the cellular substrate of GM injury was the analysis of the effects of intrauterine hypoxia-ischemia on the fetal sheep (103). Although it can be debated whether hypoxia-ischemia is particularly relevant to the majority of cases of EoP (16), this team used cutting edge combinations of high-field MRI and detailed neuropathological assessments of cell number and structure to reveal novel insights into brain injury. The team found that overall reductions in GM volume were not precipitated by neuronal cell loss, but that there were frank changes in dendritic arborizations [length, number, intersections; (103)].

Sheep models of moderate inflammatory injury are also providing important information. Exposure to intra-amniotic inflammation prior to preterm birth, was used to reveal the evolution of *in utero* inflammatory brain injury (138). Interestingly, sheep were all exposed to inflammation on the same gestational day, but sub-groups were culled every 24 h providing a detailed time course of events. Cleave caspase-3 positive cells were increased in number in the hippocampus at 2, 4, 8, and 15 days following LPS exposure, and in the cortex (at 8 days only), along with increases in MAP2 staining in both GM regions. Strikingly, there was only moderate WM injury (few cleaved caspase-3 positive cells, microgliosis, and mild demyelination) suggesting that neurons may be vulnerable to injury in the absence of overt WM damage. Reduced cortical neurons were found in another study exposing the developing sheep to LPS (139), 7 days after a single LPS challenge. In these experiments there was no difference in either astrogliosis or microgliosis at the time point analyzed compared with the previous sheep study (138) in which microgliosis was present, but astrogliosis was not.

Rodent models are by far the most common for studying potential neuropathology of EoP. In a rat model of inflammatory exposure (maternal LPS exposure at the end of gestation), significant post-natal reduction in brain and body weight were observed, and a small increase in cell death in the striatum and germinal matrix (140). In a milder injury model of prolonged induction of systemic inflammation [using systemic IL-1β exposure; (52)], there was no gross body weight change, no evidence of caspase-3 positive dying cells or alteration in the number of NeuN-positive neurons in the neocortex. However, in this injury model, there were numerous changes in gene expression for synaptic and neurotransmission related genes (141). In this same animal model, and similar to data reported in human cases, a specific alteration in interneuron number was identified in the neocortex (42)—a finding supported by a number of other early-life inflammatory exposure models (142, 143) and preterm birth models (4). It is likely that the migration and differentiation of these cell populations is affected, though many studies show that injury reduces or repairs in adult mice, following early-life inflammation (42, 143). The important advantage of rodent models is the potential for behavioral testing, where aspects of human clinical disease can be recapitulated. In the inflammatory injury models just described, behavioral dysfunction has been reported, including reduced social interaction (143), short and long-term memory deficits (46, 52), attention-shifting deficits, and anxiety-like behavior (142). These behaviors are commonly found in preterm infants, as described above, and in other NDDs, thus supporting the face validity of these models. This is further supported by an extensive body of work showing reduction in GABAergic interneurons or expression of parvalbumin (as distinct from a reduction in cell number) in clinical ASD cases (144–146) and genetic models of NDDs (147–149).

## EoP as a Synaptopathy

A synaptopathy is a disease or disorder caused by dysfunction of synapses. This dysfunction can arise due to a mutation in a gene encoding a synaptic-related protein, such as an ion channel, a neurotransmitter receptor, or a protein involved in neurotransmitter release; alternatively, a synaptopathy may be due to structural deficits in extension of neuronal arbors and synaptic process. Whether EoP can be defined as synaptopathy requires further study, but we suggest that this is likely to be an important part of the neuropathology of this condition. The changes in EoP of gross GM volume changes, variations in growth rate, and patterns of cortical folding discussed above all reflect a combination of microstructural deficits (150) and connectivity (97, 151, 152) including delayed acquisition of the default mode network, as assessed by MRI techniques. Additionally, there is the fact that EoP predisposes to strikingly increased odds of a diagnosis of a NDD that are clearly recognized as synaptopathies, such as: ASD, up to 17-fold increased rates (9, 153); attention deficit disorder, up to 2.5-fold increased rates (154, 155); epilepsy, up to 5-fold increased rates (156, 157); and decreases in IQ directly proportional to the severity of their preterm birth (158, 159).

Considering the developmental events happening during the period of preterm birth, it may be expected that alterations should be found in patterns of neuronal migration, time frames and degrees of arborization, axon extension, and synapse formation. On this subject, the recent study by Petrenko et al. (160), provides a number of important insights. In a highly reductionist model of selective neuronal apoptosis in layer 5 of the cortex, induced by diphtheria toxin (161), the authors showed a progressive loss of ~20% of neurons within the cortex over a 14-days period. While this degree of neuronal loss is unlikely to occur in EoP, the pre- and post-apoptosis changes to the brain have interesting correlates for the injury observed in EoP. Specifically, there was an associated increasing presence of astro- and microgliosis, retraction of dendritic arbors in dying neurons (days 3–5), and increased arborization (branch number and length) in the surviving neurons [day 14; (160)]. Alterations in dendritic arborization have been found in the GM in a number of experimental studies, many of which have already been referred to above [e.g., (103, 138)]. Additionally, a model of intrauterine growth restriction in pig, initiated at 100 days of pregnancy and assessed 22 days later, showed a loss of MAP2 staining in the parietal cortex and hippocampus, which was interpreted as disrupted somatodendritic neurites (162). Intrauterine growth restriction is an important contributor to poor perinatal outcomes, particularity in preterm born infants [see (163)]. Reduced dendritic branching and spine immaturity have also been reported in the CA1 region of hippocampus in a model of preterm birth in rabbit kits (30) and in the granular layer of the dentate gyrus in a maternal inflammatory activation (using i.p. poly I:C exposure) model in mice (164). These assessments are harder to perform in neuropathology on clinical samples, though reduced dendritic complexity (branch number and length) have been described for somatostatin and neuropeptide Y-positive neurons in the subcortical WM of preterm born infants with WM injury (42). Dendritic arborization, and relatedly, synapse formation [something also disrupted in these models; (42, 102, 165)], are essential developmental events for ensuring appropriate connectivity in the brain, and disruption in these processes have been implicated in a number of functional disorders of the brain (discussed below). The vulnerability of synapse structure in preterm born infants is clearly shown in a study that revealed a relationship between brain injury in preterm born infants and single nucleotide polymorphism (SNP) variants in the gene for the post-synaptic protein 95 [PSD-95, *DLG4*; (166)]. This work focused on a novel role for PSD-95 expressed specifically by microglia in early development in EoP, but the patient SNP data also suggest a wider vulnerability of synapse structure in preterm-born infants.

While evidence of EoP as a synaptopathy inevitable comes from neuropathological studies, our best capacity to clinical recognize disease, stratify patients for treatment, and monitor progress comes from neuroimaging. When relating *in vivo* imaging to pathology, the study by Petrenko et al. (160) suggested that (a) neurons loss could be detected by decrease in N-acetylaspartate and N-acetylaspartylglutamate and astrogliosis with reduced Glutamate/Glycine ratio, using magnetic resonance spectroscopy within 3 days of injury; and (b) diffusion MRI could also detect microstructural injury within 3 days of cell death induction, starting with increased water diffusivity (mean diffusivity) and extending to reduced factional anisotropy (FA) due to altered dendritic arrangement. Ball et al. (150) have shown a developmental decrease in FA in the cortex over the preterm period, with preterm born infants lagging behind term born infants in this maturational process, i.e., with a higher FA at term equivalent age. Modeling by Dean et al. (103) supports the idea that this increased FA value is due to delays in the normal dendritic arborization of the cortical neurons over this period. Vinall et al. (167), studied variation in diffusion MRI values between two scans in a cohort of very preterm infants. Their work showed that increased FA in the cortical GM at scan two was independently associated with reduced gestational age, birth weight, and slow weight gain. In addition, changes in FA were related to the second and third eigenvector direction, rather than the primary eigenvector direction. Collectively, these data imply that delays in cortical maturation were most likely driven by delays in neuronal process formation, or cell loss, and that cortical maturation was associated primarily with the phase of neonatal growth (167). Structural connectivity studies, typically based on the integrity of WM tracts using diffusion MRI, have shown a topographically dependent timetable of connectivity developing brain, which is impaired in the preterm brain (168, 169), and which is altered in nature over time, but persists in some form to adulthood (170). While these measures are not directly assessing cortical GM injury, it is likely that an interplay between WM and GM development occurs and that altered connectivity maps will reflect changes in GM development. These structural alterations are also likely to have functional consequences that reflect both local and global connectivity.

## Interplay of Structural and Functional Deficits in EoP

Altered structural and functional connectivity can be identified in the brains of preterm infants at term equivalent age, using combined diffusion and functional MRI (171). Aside from studies testing passive function, including touch and auditory stimulation, the majority of functional MRI studies in preterm infants have investigated resting states. Collectively, these resting state studies suggest that there is modular organization of the connectivity of the preterm brain, as is seen in the mature brain, but that integration between networks is altered (172–174). In these studies, there is evidence for disruption in both cortico-cortical and cortico-subcortical networks (172, 174), and reduced connectivity between areas associated with motor function, cognition, language, and executive function (173).

The electroencephalogram (EEG) is a clinical tool that has been shown to have some potential to monitor and predict severity and outcome of EoP. EEG waveforms are immature in the preterm brain, but appear to have some characteristic changes that can be used as a biomarker, including seizures, EEG suppression, and mechanical delta brush activity (175–178). The rate of spontaneous activity transients on EEG in preterm born infants with or without GM-IVH, measured over the first 48 h of life, was associated with cortical GM volume growth, increased gyrification index, and increased FA in WM tracts (179). Additional studies of the association between early EEG and cortical growth have revealed very specific band frequency relationships and with spontaneous activity transients (SATs) (180). As we begin to understand the biological drivers of these events, it will provide further information of the structure function relationship of the EEG recordings. Whitehead et al. (181), using EEG, showed that gross injury initially disrupts signal recruitment from cortical circuits. Signal recruitment appears to eventually be reinstated following injury but remains different from individuals without gross injury. Importantly, EEG abnormalities assessed shortly after birth (a week to a month after birth) were able to predict both developmental delay and cerebral palsy at 18–24 months (182, 183).

In animal models, fMRI has not been used, but EEG has been used extensively in sheep models of *in utero* hypoxic ischemic injury (more closely modeling hypoxic-ischemic encephalopathy) and shows reduced maturation of the EEG signal over time, seizure susceptibility, and microscale epileptiform events in the latent phase (up to 7 h post-injury) prior to seizure onset that correlates with cell death (184, 185). Following intrauterine inflammatory exposure in fetal sheep, changes in developmental patterns in alpha and beta power (reduced) and delta power (increased) have also been reported (186). However, while there is widespread evidence of altered EEG parameters in both clinical and preclinical studies, it is not clear how well these changes related to the neuropathology and how predictive they are for outcome. This work is only just beginning in clinical populations [e.g., (181–183)], but in pre-clinical studies, a number of studies have found a disconnect between EEG results and activity and arousal (187) or neuropathology (188, 189). However, it should be noted that the pathology examined in the study by Galinsky et al. (189) was largely focused on WM, rather than GM, features, and therefore may provide a limited understanding the pathological correlates of EEG. Van den Heuij et al. (185), for instance, have reported improved EEG findings together with reduced cortical and deep GM damage following intrauterine artery occlusion in the fetal sheep. In rodent models, EEG studies are less common, due to the size of the post-natal brain. Using *ex vivo* multi-electrode arrays, Mordel et al. (190) showed that inflammation and hypoxia, alone or together, increased the excitability of cortical neurons, in a glutamate receptor dependent manner. Interestingly, this research group has also shown that inflammation-induced alterations in cortical neuron spontaneous burst activity subsequently results in an increase in apoptosis in the same cell population (191). The work of Mordel et al. (190) suggests that altered electrical activity in the cortex occurs only in the first few weeks after injury and that it recovers in adulthood. However, long-term alterations in spontaneous and mini-inhibitory post-synaptic currents, a more subtle measure of neuronal activity, was found specifically for parvalbumin-positive interneurons following fetal exposure to inflammation (142). Electrophysiological studies in the preterm sheep have shown altered excitability in subplate neurons (133), as well as reductions in intrinsic excitability, altered polarization dynamics and reduced long-term synaptic plasticity in the hippocampus, following hypoxia-ischemia and hypoxia alone (136).

The relationship with structure and function is complex and needs to be understood better at a (sub-)cellular level in the context of EoP. However, the study of Zaslavsky et al. (192) in iPSCs from ASD patients shows increased dendritic arborization and synaptic connectivity associated with a significant increase in sESPC frequency, supporting suggestions that altered neuronal morphology does change cellular function (rather than being compensated for in the function of the cellular communications pathway). This link between structure and function, the capacity for one to affect the other, and the plasticity for recovery is a particularly important point to consider when exploring new therapeutic targets, and optimal periods of treatment, for EoP and NDDs. This concept has recently been supported in a study of genetically encoded epilepsy, where timely treatment with Bumetanide altered long-term neuronal activity and network formation (193).

## Potential Therapies for EoP

### Gray Matter Targets

The most obvious change in the GM of preterm born infants are reductions in volumes on MRI, changes that persist with increasing age. These gross changes are likely mediated by limited but significant cell death, changes in sub-classes of interneurons, and, across neuronal classes, reductions in arborization and/or synaptic number. There are no therapies designed to target GM injury in the preterm specifically. Given that there are striking similarities between the GM changes in EoP and NDDs, it would seem appropriate to consider if any therapeutic candidates from the NDD field might have efficacy in EoP. Current pharmacological strategies for the treatment of ADHD focus on normalizing, but not repairing, disturbances in synaptic transmission and activity (194), and the same is the case for the various forms of epilepsy (195, 196). For ASD, therapy focuses on treating the symptoms of the disorder, such as risperidone, to reduce irritability via antagonism of central type 2 serotonergic (5-HT2) receptors and central dopamine D2 receptors (197). There are no therapies for ASD to treat the underlying deficits in social abilities. Other NDDs, such as intellectual disability and learning disorders (dyslexia and dyscalculia), together with ASD and ADHD, are successfully treated with behavioral interventions. It is believed that these therapies do rewire the brain (198), but whether they are capable of repair is not at all established.

A recent review of the drugs under investigation review for ASD highlighted that potential therapies fall into several clear classes—GABA/glutamate modulators, neuropeptides, immunologics, and dietary supplementation (199). The only therapies whose specific underlying premise is to permanently alter the structure of the brain are immunological therapies; although, neurotransmitter modulators given at the optimal stage of development may normalize aspects of structural and functional development—something that needs to be considered in future research. That immunological therapies might enable repair is based on the underlying idea that, in the brains of people with ASD, there is a persistent immunological dysfunction that itself is the cause of the core social deficits. As such, removing this dysfunction allows the brain to return to a normal structural and functional state. A very similar process of persisting and damaging inflammation is hypothesized to occur in the brain after perinatal brain injury (200) that evidence begins to accrue, which, in this context, it is also a valid therapeutic target (57, 201).

Another exploratory area of understanding and treating ASD and other NDDs is the gut–microbiome–brain axis (202, 203). Gene mutations associated with autism pathogenesis impair brain and gut function and contribute to core and comorbid symptoms reported in autism (204, 205). The gut and brain share cellular structures, molecular pathways and processes that likely cause shared vulnerability to processes leading to autism (203). For instance, gut and brain synaptic structure and function are similarly vulnerable to disturbances in structural proteins, such as neuroligins, post-synaptic density proteins, and Shanks (166, 206–210). An inexorable production of gut microbe-derived neuroactive metabolites influences gastrointestinal function, and these also traverse the BBB to exert potent effects on the brain (211–213). Importantly, microbiome-mediated gut and brain crosstalk even alters early brain development (214, 215) via dysbiosis, which impairs the function of the brain's chief “building managers” and resident immune cells—microglia. Microbe-derived metabolites also regulate the function of the BBB itself (216) demonstrating the integral nature of the microbiome-gut-brain axis in brain health. As such, research investigating factors modulating the gut–microbiome axis in NDDs may uncover novel mechanisms for treatment (217, 218).

Considering the options from the classes of drugs already being tested in models of EoP, we find that, despite many compounds being tested (with mixed results), most have not considered outcomes in the GM. There are some notable exceptions, such as MgSO_4_ pre-treatment in a rat model of preterm HI (modeling antenatal treatment in at-risk mothers), significantly reduced tissue loss in the hippocampus and striatum and were associated with reduced neurological injury score (219). MgSO_4_ has also been tested in a sheep model of perinatal asphyxia, reporting reduced seizure burden, but worse WM outcomes and no GM neuropathology (189). Clinically, MgSO_4_ has a number to treat of 54 (220), though due to the nature of pre-treatment of at risk individuals, the exact efficacy is difficult to determine; a Cochrane review of four trials of antenatal treatment of at-risk women showed no significant effect on mortality or neurological outcome (220). Erythropoietin in this environment has not been shown to be protective for qualitative WM or GM injury when administered as three doses of 25 μg/kg within the first 2 days of birth in preterm infants (221). This is despite positive GM outcomes in rodent (188, 222) and sheep models (223). Robinson et al. (222) showed that 2,000 U/kg erythropoietin (~17 μg/kg), administered post-natally following intrauterine occlusion, was beneficial for both WM and GM, ameliorating behavioral deficits in gait and social interaction and fractional anisotropy changes in the WM, hippocampus, and striatum. In their study of perinatal injury, hypoxia-ischemia in the post-natal day-3 rat, van de Looij et al. (188) showed that erythropoietin improved somatosensory-evoked potentials and diffusion parameters in the WM, when measured with MRI, but didn't prevent cortical tissue loss. Wassink et al. (223) assessed neuronal number and cell death in the caudate, showing a significant improvement with erythropoietin (5,000 IU loading dose, followed by 832 IU/h) in the preterm sheep, as well as reduced seizure burden. More positive data on erythropoietin have been found for WM injury [reviewed in (79, 224)], supporting the numerous on-going clinical trials for this drug; however, it is clear that additional therapeutic agents need to be tested for GM efficacy.

## Links Between EoP and Neurodevelopmental Diseases

It has been unequivocally established that preterm born infants have increased rates of diagnosis for NDDs, including ASD, ADHD, and generalized learning disorders (5, 7–9, 225). It is also clear that, in the brains of people who suffered from EoP and those with NDDs (and from their matched preclinical models), there are a striking number of shared pathomechanisms. In this section, we will highlight key phenotypic, macrostructural, genetic, cellular, and sub-cellular processes shared with EoP and in cases of NDD. We will focus on the GM; but, we wish to highlight that for the WM these links between EoP and NDD are more established, such as shared deficits in corpus callosum structure in people after EoP and those with ASD and ADHD (226).

Recent work has assessed in detail the specific characteristics of behavioral disturbances in people born preterm with NDD, compared with people born at-term with an NDD [see reviews (227, 228)]. In general, in preterm vs. term NDD, the phenotypic presentations are similar. However, there are important differences. For instance, in people born at term, there is a higher rate of ADHD in males compared with females; but, this sex difference in not observed in people with ADHD who were born preterm (229). For ASD, a greater proportion of preterm (vs. term-born) males reported comorbidities (sleep apnea, seizure disorders, and ADHD) and people born preterm (particularly females) were more often non-verbal (230). Another recent small study of children with ASD demonstrated that, compared to term children, the preterm children had higher quality peer relationships and socioemotional reciprocity, but poorer non-verbal behaviors that regulate social communication (231). None of the current literature has indicated a problem with diagnosing those born preterm using the current diagnostic criteria. However, we speculate that just as autism has been expanded and refined into a complex spectrum of disorders that, in the future, ASD phenotypes specific to preterm born infants may be defined.

With increased MRI analyses of the GM in individuals with EoP, we begin to see a clear pattern of similarities in changes in brain structure in people with ASD and those born preterm—there are shared changes in the orbitofrontal regions, the amygdala, the basal ganglia, the hippocampus, and the cerebellum [reviewed in (227, 232)]. There is also a parallel with the altered cortical growth in preterm born infants and equivalent findings in ASD and ADHD patients. In MRI studies of ASD and ADHD, decreased GM volume has been associated with both conditions (233–237). In ASD, decreases have particularly been found in areas related to social behavior networks (233, 235, 237), while regions associated with inhibitory control (234) were changed in ADHD. In both cases, it is clear that patterns of GM deficits alter through the disease course (235, 238). Changes in the volume of GM in preterm infants/children/adolescents have been found in many of these regions [e.g., (91, 92, 239)], but are typically more widespread. Variation between studies has, of course, been reported, with not all studies finding cortical GM volume changes or associating them with neurological outcome. However, these are in the minority, and it has been suggested that these may be due to difficulties in accurately recognizing the gray-white matter boundary in the developing brain (240). Interestingly, in addition to this overlap in affected brain areas in both EoP and NDDs, MRI studies are also showing alterations in cortical networks [e.g., (236, 241)] in ASD and ADHD that warrant further exploration, and may come from as similar anatomical basis as in the EoP studies.

A newer avenue to link EoP and NDDs are genetic studies, such as genome-wide association studies (GWAS), copy number variant (CNV) studies, SNP, and haplotype studies, and these are revealing common risk factors. For instance, we have recently uncovered that an SNP in the gene for PSD95 is associated with poorer outcome for preterm born infants (166), mentioned above, as genetic variation in polymorphisms for PSD95 is a known risk factor for ASD (242). Common genetic variants and methylation patterns have been revealed in focused studies of people with ASD, with and without prior history of preterm birth (243). Changes uncovered by these targeted studies include tyrosine-protein kinase Met (*MET*), Neuregulin 3 (*NRG3*), and serotonin transporter (*SLC6A4*). A great deal can also be learned from comparing findings from studies of NDDs and studies of prematurity and EoP. For NDDs, there are numerous genes associated with synapse formation identified from GWAS studies including Shanks, Neuroregulin, Neurexin, and Contactins [reviewed by (244, 245)]. Many of these genes also associate with preterm birth or outcomes after preterm birth. Of note, neuregulin is found associated with infant outcome, with polymorphisms increasing mRNA levels in patients associated with better outcomes in babies born preterm (246).

A key vulnerable neuronal subpopulation in EoP is interneurons, although it is still unclear which populations are the most vulnerable at which time point and in what regions based on the human and preclinical studies (4, 42). Research into neuropathology in NDD, via post-mortem studies and animal models of NDD, also conclusively illustrates changes in interneurons (146). Quite strikingly, in a synaptic protein knockout model of ASD (PTEN KO), interneuron transplantation rescues social behavior deficits (247). This study also questions the established idea that interneuron deficits associate with NDDs due to negative effects on inhibitory circuit activation (248), as, although interneuron transplantation rescued the behavioral phenotype, there were no improvements in circuit function.

No discussion of the similarities between NDD and EoP could be complete with highlighting the shared common pathological process of neuroinflammation, which has, at its core, the aberrant activation of microglia. Indeed, across NDD and EoP models and human studies, evidence shows that microglial activities are altered [thoroughly reviewed in (22, 249–251)]. A chief function of microglia during development, but also throughout life, is regulation of connectivity via refinement of synaptic number [(252–254); and reviewed in (255–257)]. Based on all the evidence for the role of microglia and the presence of inflammation (both systemic and central) in EoP and NDD it is clear that microglia (and their effects on synapses and neurogenesis) are an important starting point in understanding GM pathology across NDDs and EoP and also a shared target for neurorepair.

We outlined above the reasons that EoP can be considered a synaptopathy, including genetic associations between injury severity and synaptic genes, connectivity deficits, and that preclinical studies show synaptic immaturity plus arborization deficits. These characteristics are also common among NDDs, and NDDs are clearly characterized as synaptopathies (258–261). For example, about half of the genes identified as candidate genes in people with ASD code synaptic proteins (262). Additionally, animal models of abnormal synaptic pruning induced by abnormal microglial function (227), or via genetic perturbation of synaptic structure (263), have cognitive and behavioral deficits reminiscent of NDDs. Thus, perhaps it is the collective change in these functional units of the neuron that give rise to the shared gross volumetric changes and pervasive behavioral problems in people with NDDs and due to EoP. Though it should also be said that a great many children and adults who were born preterm and who had EoP have typical neurodevelopmental profiles, potentially and interaction of genetics and environmental challenges in these case lead to structurally resilient synapses. There is clearly need for a better understanding of the vulnerabilities leading to NDDs and negative consequences after EoP.

## Conclusions

Imaging and neuropathological studies indicate changes in GM are a subtle but substantial contributor to EoP. The full nature of this injury is probably only just being discovered and would benefit from more longitudinal MRI studies, with closer integration of both patient genetics data and neuropathology where possible. Given the link between GM injury and long-term cognitive and behavioral disorders, it is important to therapeutically target this injury, distinct from the WM injury aspects of EoP. In particular, while preterm birth and EoP increase the risk of NDD in later life, the current evidence suggests that preterm born infants may make up a specific subset of cases in these disorder spectrums and could benefit from a distinct treatment paradigm. In terms of what this therapeutic paradigm might look like, it is likely that a combination of ameliorating (e.g., anti-inflammatory or growth supporting) agents and restorative agents (e.g., drugs facilitating normal structural-functional development) will be required. If these treatments are delivered at optimal periods of brain development, it may be possible to limit the need for life-long symptom controlling medication. In this regard, it is necessary to focus more research on the synaptopathic aspects of EoP. Current research in this area is only the tip of the iceberg, particularly lacking in clinical studies, and increased understanding of the injury mechanisms and plasticity during the post-natal period may identify new therapeutic targets. Our great hope is actually that this proposed work becomes redundant. We hope that our highly skilled and motivated counterparts working on prediction and prevention of preterm birth have major breakthroughs. However, pragmatically, even major breakthroughs will take decades to make it across high and middle/low economic settings, meaning that millions more babies are going to need us to better understand the GM and its changes after EoP.

## Author Contributions

All authors listed have made a substantial, direct and intellectual contribution to the work, and approved it for publication.

## Conflict of Interest

The authors declare that the research was conducted in the absence of any commercial or financial relationships that could be construed as a potential conflict of interest.

## Abbreviations

ADHD: attention deficit hyperactivity disorder
ASD: autism spectrum disorder
EEG: electroencephalogram
EoP: encephalopathy of prematurity
GM: gray matter
FA: fractional anisotropy
IVH: intraventricular hemorrhage
MRI: magnetic resonance imaging
NDD: neurodevelopmental disorder
PVL: periventricular leukomalacia
WM: white matter.
